# A Case Where an Artificial Plate with False Teeth Was Accidentally Swallowed, Dislodged, and Ultimately Passed by the Patient

**Published:** 1871-06

**Authors:** Henry Smith

**Affiliations:** Assistant-Surgeon to King's College Hospital.


### ARTICLE X.

### *A Case where an Artificial Plate with False Teeth was Accidentally Swallowed, Dislodged, and Ultimately Passed by the Patient.*

By Henry Smith, F. R. C. S.,
Assistant-Surgeon to King's College Hospital.

The interesting cases of lodgment of artificial teeth by Mr. George Pollock, and the extraordinary instance of the passage of a gold pencil case through the intestinal canal reported by Sir William Fergusson in the pages of *The Lancet*, must be familiar to the profession. To these I have now to add a case of much interest, where a very ugly foreign body, was got rid of safely, partly by the efforts of surgery and partly by those of nature.

On the night of November 30th, 1870, I was summoned to Mitcham by Dr. Hamilton, who had the wisdom to telegraph the nature of the accident; and I thus went down with suitable instruments. I found a corpulent butcher in great distress, he having six hours previously, by some means or other, allowed his false teeth, with their plate, armed with most formidable hooks on either side, to slip down his throat. He at once sent for Dr. Hamilton, who, on passing his finger down into the pharynx, could distinctly feel the foreign body on the right side; but, unfortunately, he had not the requisite instruments, and in his endeavors to dislodge it, the body got out of his reach.

On my arrival, the patient pointed to just above the clavicle on the right side as the spot where the intruder lay. I at once passed a long pair of crane-bill œsophagus forceps, and imagined I could feel the foreign body, but I could not catch it. I tried carefully again and again with other instruments; but, as considerable bleeding ensued, and as there was great distress on the part of the patient, I determined to push the body down into the stomach, and with that view passed a full-sized œsophagus bougie into the stomach, when the sensation of the presence of the tooth-plate at once ceased.

The patient was ordered to keep perfectly quiet, and to take plenty of gruel porridge and oil. He had no pain at all except for about five hours on the day the foreign body passed away, which event happened nine days after I had pushed it down. The patient has suffered nothing since.

I am aware that it is a dangerous practice to adopt the course I did in this case. I know of two instances where death followed this plan of treatment, one of them from hæmorrhage; but I am not sure that prolonged and forcible attempts to extract such a formidable-looking body would not be attended with as much danger; and there are so many instances on record where such ugly bodies have passed through the intestinal tract with safety, that the surgeon is quite justified in resorting to the expedient I adopted, providing he has first made an effort to extract the substance. The attempts I made were quite sufficient to tell me that I should not succeed in extracting the false teeth; and, indeed, I necessarily put the patient to so much pain and distress, and brought about so much bleeding, that I was only too glad to desist from further attempts. If these attempts do not succeed at once, they are not likely to succeed at all. Remarkable cases have occurred where such foreign bodies have been extracted, and, among others, one happened in the practice of Dr. Vine, who succeeded in extracting with a probang a plate armed with seven false teeth, an inch and three quarters in length, and which had become engaged in the lower part of the œsophagus.—*London Lancet.*

